# Supplementary material for: Meta-Analysis of Human and Mouse Biliary Epithelial Cell Gene Profiles
Source: Cells. 2019 Sep 20;8(10):1117. doi: 10.3390/cells8101117 (PMC6829476; doi:10.3390/cells8101117)
Supplement: Supplementary file 1 [file cells-08-01117-s001.pdf]

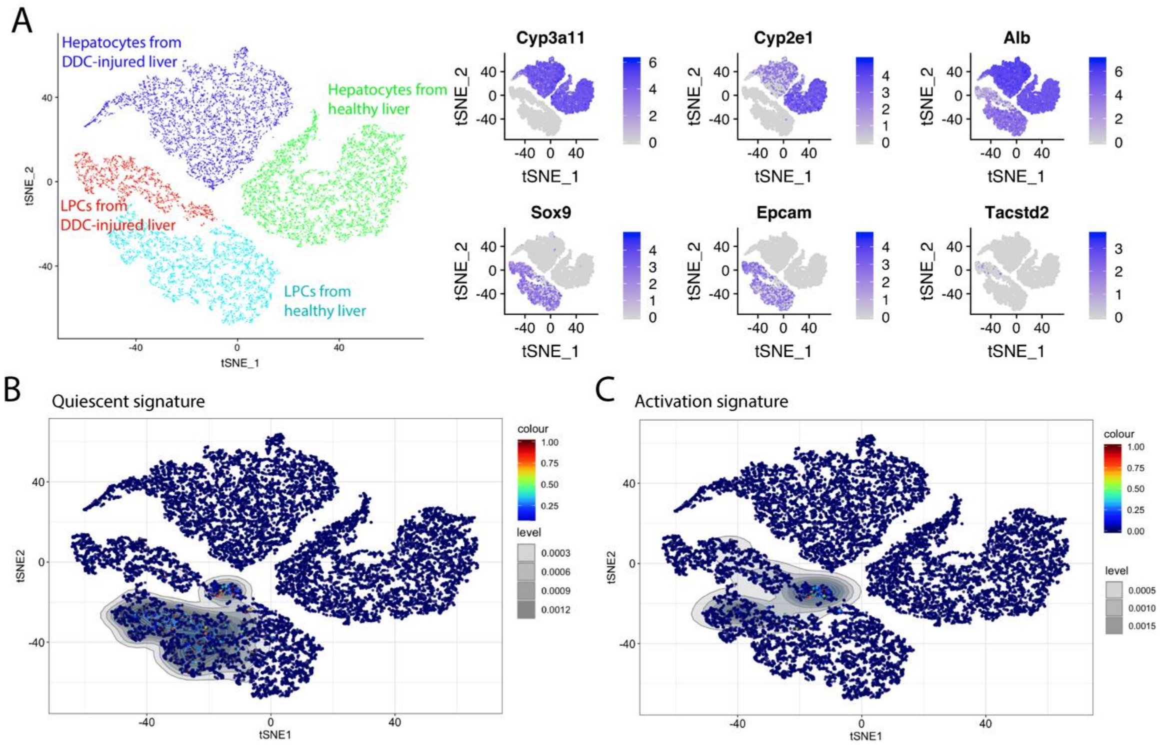

Supplementary Figure S1. (A) TSNE plots of scRNAseq data of hepatocytes and LPCs isolated from healthy and DDC-injured livers, validated hepatocyte (Cyp3a11, Cyp2e1 and Alb) and LPC (Sox9, Epcam, Tacstd2 aka TROP2) markers. The blue scale bar represents normalized counts. TSNE plot by single cell signature explorer with single cell signature analysis of quiescent (B) and activation signature (C) on single cells from Pepe-Mooney et al [28]. Signature scores are presented for each cell using colour gradient and grey colour overlap shows the location of most cells with a high signature score.

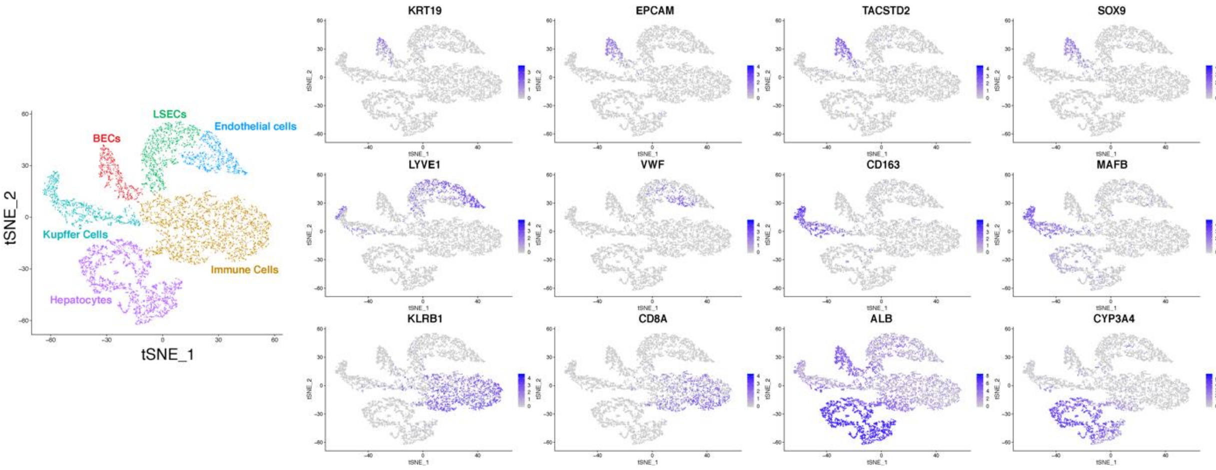

Supplementary Figure S2. TSNE plots of scRNAseq data of different cell types isolated from healthy human livers, validated hepatocyte (ALB and CYP3A4), BEC (KRT19, EPCAM, TACSTD2, SOX9), LSECs (LYVE1), Endothelial cell (VWF), Kupffer cell (CD163, MAFB) and other immune cell (CD8A, KLRB1) markers. The blue scale bar represents normalized counts.

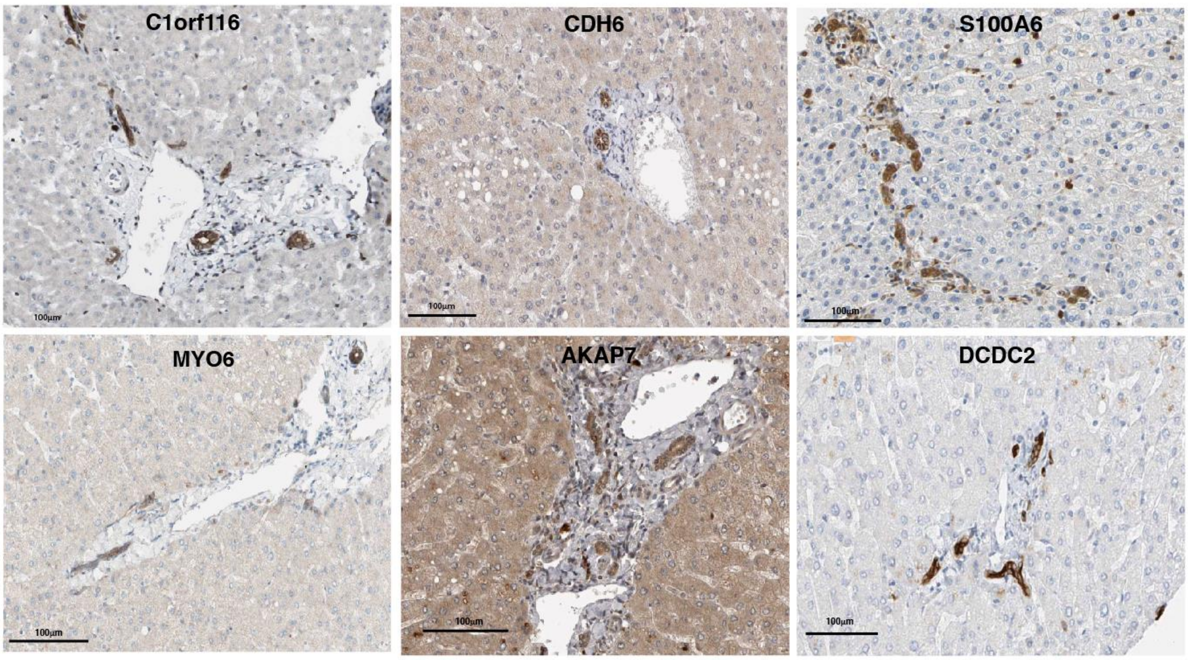

Supplemental Figure S3. Immunohistochemistry staining of new potential BEC markers from our BEC signature, obtained from protein atlas.

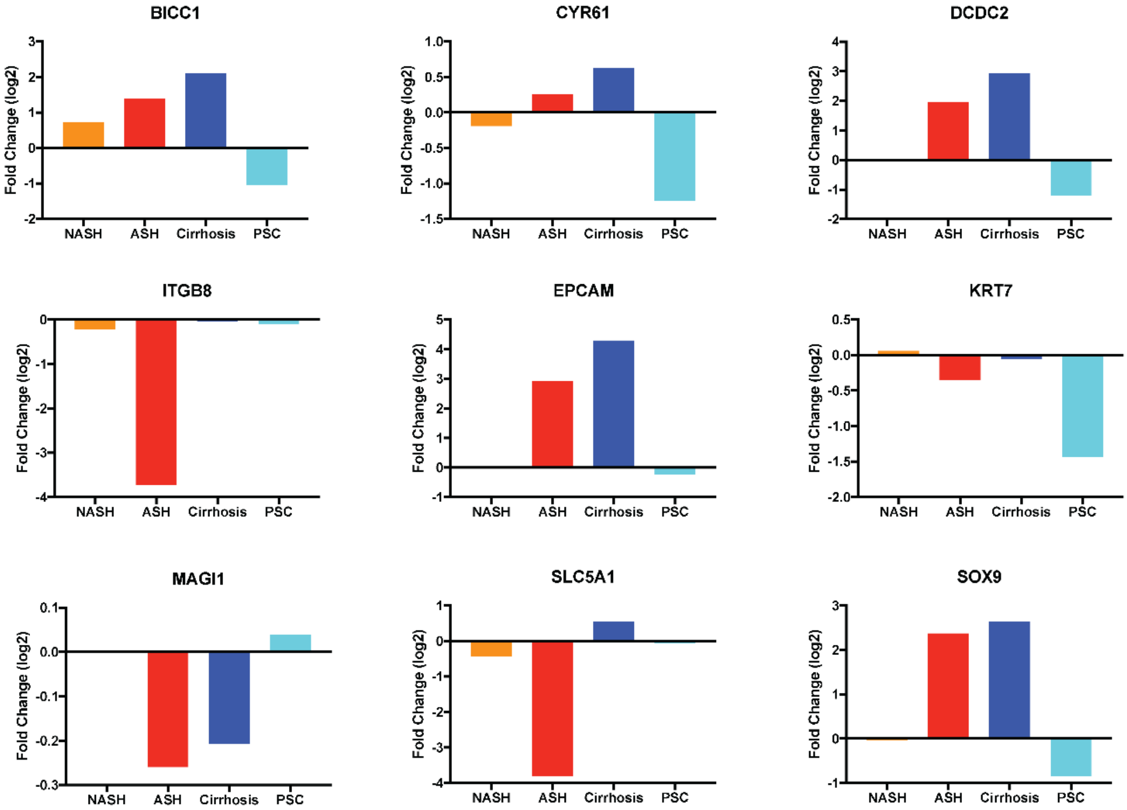

Supplemental Figure S4. Gene expression of genes in the scBEC signature on transcriptomic data of NASH, ASH, Cirrhosis and PSC. Expression values are compared to their control samples (healthy livers).

Supplementary Table S1. BEC labeling based on injury model.

BEC marker used for isolation	Injury model	Specie	BEC Labeling
MIC1 1C3	Healthy	Mouse	Quiescent
MIC1 1C3	DDC injury	Mouse	Activated
HNF1b	Healthy	Mouse	Quiescent
HNF1b	DDC injury	Mouse	Activated
HNF1b	CDE injury	Mouse	Activated
Folx1	Healthy	Mouse	Initiation
Folx1	DDC injury (3 days)	Mouse	Initiation
Folx1	DDC injury (7 days)	Mouse	Initiation
Folx1	DDC injury (14 days)	Mouse	Activated
EPCAM	Healthy	Mouse	Quiescent
LGR5	CCl4 injury	Mouse	Activated (not used for BEC activation signature)
EPCAM	Cirrhotic liver	Human	Activated
SP	Cirrhotic liver	Human	Activated
TROP2	Cirrhotic liver	Human	Activated

Supplementary Table S2. List of genes expressed by only human or mouse BECs.

Gene list Venn diagram Fi					
Only in human LPCs				Only in mouse qLPCs	Only in mouse aLPC
HYAL4	SFTA2	NBEAL2	TOM1L2	THSD7A	RPUSD2
MT1IP	SGMS2	TNFRSF12A	PIEZO1	FLRT2	PHF14
DAZ2	RASEF	B3GNT7	CAPN6	OSMR	ANO1
LINC00311	SLC9A4	VEGFA	SYDE2	CAV1	CKMT1A
ANXA8	MIR17HG	NCK2	PURB	GJA1	BTC
LUZP2	AP1M2	CBX6	WWP2	FBLN2	LRRTM2
MIR663A	TNFAIP2	ZNF295	INO80D	ARHGAP6	MRPS11
LOC100132526	MAP3K8	PPP4R1L	DDX26B	DSEL	TNKS
LOC440970	BTG2	PAPLN	BACE2	HS3ST1	TMEM106B
MIR3064	LOC90834	ADCY6	ATP9A	ANK2	SMPD4
SLC13A1	C14orf43	KLHL11	TGIF2	CBR3	SRGAP2C
NPPA-AS1	NR4A1	ARL4C	DOCK5	LIMCH1	CAPG
HSPA6	RMRP	SLC41A1	KLF7	ADAMTS15	ZNF274
HIST2H2BA	KLF2	LOC389641	PFKP	ITIH5	CIB1
LOC284865	GPRC5B	AGAP8	TUBGCP6	WTIP	SETD7
ARC	NSUN7	ARHGEF18	FEM1C	KAZALD1	POT1
HSPA7	RASAL1	POMT2	SBF1	WNT5A	EIF2AK3
SNORA4	SFRP5	GRAMD1B	HIVEP1	RHOJ	ST6GALNAC2
TNFSF9	CCDC148	TLE4	TLE2	ANKRD29	SERF2
OR7D2	POTEM	CHORDC1	LOC652276	MATN2	NFIC
KIAA1875	CDS1	JMJD6	FAM46C	PRSS23	CDKL2
GSG1	SPTB	ANKRD36	CROCC	PCDHB15	C17orf97
HSPE1-MOB4	SYCE1L	FCHSD1	IQCK	SERTAD4	SHISA2
RNF222	WWTR1-AS1	TRIM62	DHX34	FSTL1	ERLEC1
LINC00485	TNC	ZBTB2	TCOF1		FRK
DUOX2	LOC728643	FAM171A1	GALNT4		ABHD2
TNF	CD83	LOC100652999	CHD7		SSR4
WASH7P	AGPAT4	MLK7-AS1	RUNX1		MYCBP
DNAJB1	SEMA3B	SMAD3	AKAP17A		PSMB1
SLC14A2	CTSL2	CTGF	ARHGAP32		NFIA
KCNA7	C2CD4B	RHBDF1	DDX51		SORBS3
BAG3	PMEPA1	HIST1H2AE	CREBBP		FAM83E
C4orf26	LAMA3	SLC25A29	RANGAP1		HNRNPL
CLDN4	CDCP1	RAB36	IDUA		HADH
CXCL3	UNC13D	PRPF40B	KLC2		ACTR2
C3orf52	CA9	STX3	HERC2P2		RCBTB1
FOSL1	FSD1L	MUC20	CLASRP		MRPL19
PCLO	ABCC1	TJP2	CAMTA2		ANO8
IQCA1	CXCL5	IRGQ	YTHDC1		SERBP1
HSPH1	GOLGA6L6	KDM2A	ZC3HAV1		SRGAP2B
VSIG1	FLJ11235	PPP1R26	SPINT2		GTF3C6
PMAIP1	PLK3	CUL7	PRRC2C		GGCT
ZC3H12A	AMY2A	LOC146880	SAFB		PRDM16
WNK2	CPT1B	FKBP4	PIGA		FASTKD3
PPP1R13L	FGF5	TSPYL2	WSB1		CDK13
C6orf141	NR6A1	LRIG3	ABL1		CAB39L
EREG	PI4KAP1	TRIM47	DALRD3		PYGO1
OVOL1	PVRL1	PLAGL2	RCOR1		SPA17
EGR4	SDK1	LOC147670	ATXN2L		CD151
IGSF3	HSP90AB1	KIFC3	EXOC6B		MARCH2
PPP1R15A	MEX3D	CCNL1	MESDC1		ZNF410
RNF103-CHMP3	PLCD3	RIC8B	FNBP4		PRKRA
ETV3	TBC1D3G	SH3RF1	HMGCR		ALDOC
OXTR	NEURL3	PLXNA3	AHI1		HNF1B
ANKRD33B	PCDP1	RBBP6	MARCH6		DAB1
LIF	FAM83B	SLC9A7	OFD1		WDR61
PRAMEF5	TMEM136	MOB3B	PRRC2A		WRNIP1
RNF39	RFX2	ZBTB40	CENPJ		B4GALT6
HSPA1L	FRMD6-AS1	ABCB1	PNPLA2		LRBA
LOC100506305	TMC4	WDR6	CHD8		SLU7
NUAK2	TUFT1	DUSP18	ATXN2		TNS3
WNT7B	TMPRSS13	ATP1A1	ZDHHC21		SIKE1
HIST2H3A	TTN	DAPK3	RALGDS		SART1
HIST2H3C	SEMA6A	RAB3IP	PPP1R15B		MAX
CHST9-AS1	KLC3	SLC9A1	PGM2L1		ST13
EDN1	MGC12916	ZMIZ2	CBFA2T2		PRR15L
OR6W1P	FAM74A3	STX1A	H2AFX		TRIM27
CTNND2	AGAP1	KLHL17	BTG3		S100A14
FAM160A1	SLC4A3	ZNF335	MFSD6		MYO1E
LOC115110	ANXA8L2	C1orf63	LOC728377		TSPAN7
CXCL1	REC8	LOC646214	RNF169		WDR92
SLC28A3	TICAM1	HIC2	PPP1R10		ARHGAP12
GPRC5A	SMARCA4	SRGAP1	DNMBP		CROT
CLCF1	GPR176	SRRM2	CCDC93		PRR15
LOC100506994	GPRC5D	FAM193B	DGKQ		HSPB11
GATSL2	DAGLA	ZNF574	KRT18		MBOAT1
PRSS22	PLEKHG6	MUM1L1	ZBTB7A		UBA1
NFKBIZ	SHISA9	CRAMP1L	TEAD1		CEBPG
EGR1	VPS37B	ZSWIM6	HSPG2		ECSCR
TRIM29	DUSP5	NMB	LOC613037		SYT7
TFAP2C	C6orf52	DLG5	KIAA0182		OCIAD1
MAP3K9	MLF1	TNFRSF10B	AKAP8L		PPT1
RHOV	C17orf96	OCLN	MAP7D1		ATP5F1A
FBXL19-AS1	PPAP2C	ILF3	LENG8		TMEM173
STK33	C2CD4C	ARHGAP26	PRRC2B		AVL9
SIX4	FOXH1	MYZAP	DYNC1H1		PGLYRP1
GOLGA7B	VIL1	PRR7	CCDC14		PHF10
FOS	MSX1	LOC645676	BCL2L11		ZNF358
AREG	DUSP1	PAQR5	RBM6		FBXW11
HIST1H2BG	HLA-J	TIPARP	ZNF281		ROR1
SGK223	ZBTB43	FOXK1	NBPF10		PSMA3
DNAJB4	MYO10	CHKA	LATS1		IL6ST
SNORA70	TTYH1	FUT4	YPEL2		KIF2A
C1orf106	IRS2	PHC1	BCOR		MYH10
HAS3	YOD1	MAML2	DCP1A		RHBDD2
C7orf53	LINC00115	LOC100271836	SVIL		SRGAP2
ZNF165	MAFG	LOC100302650	C10orf12		ARL1
TMEM171	KIAA0556	COBL	KCTD13		B4GALT3
RRAD	LOC729603	BAZ1A	MARCKSL1		SERPINB6
EGOT	SH3RF2	THBS1	C17orf63		BCHE
RAP1GAP2	TES	SNIP1	VPS53		LYPLA1
PRDM11	MICAL3	IFRD1	SLC19A2		RPRD2
DOT1L	UBC	ISYNA1	BAZ2A		TMEM130
JUN	BCORL1	OTUD7B	SLC5A3		STT3A
ESYT3	NEDD9	AGAP7	CLEC16A		PFKFB2
SLC9A3	ZNF142	CHRM3	CTNND1		N4BP2L2
KCNMB3	DNAJA1	ZMIZ1	LUC7L		ERBB4
CASZ1	SLC45A4	CCDC11	MICALL1		RNF180
SLC7A5P2	ALPK3	RGPD3	PHIP		ARHGEF17
GUSBP11	NPC1	ENAH	RNF213		FUT2
OTUD6A	LOC100188947	EHD1	ATP11A		AEBP2
ANKRD18B	GTPBP1	CBX4	NKX3-1		MTSS2
ZSWIM4	SPTBN5	CD24	C7orf13		CD9
CXCL2	SLFN13	DSG2	SLC35E2B		CXCL16
BHLHE41	HSPA8	ARAP2	ZCCHC8		CELSR1
REM2	LPAR2	AHSA2	ZNF554		DEGS2
C11orf41	SCRN1	GPR56	PNISR		DNM1L
LRRN1	UCKL1-AS1	SERTAD1	ARHGEF5		PRCP
DNAJA4	SLC29A2	RARG	VPRBP		LYNX1
EPPK1	LOC389023	CHMP4B	RAB11FIP5		ARHGAP29
FAM189A1	TNFRSF10D	AZI1	RASAL2		LRP5
TNFAIP3	SCD5	MLLT3	MYBBP1A		VPS25
GREB1	ARSJ	CHTF18	MARK2		HTATSF1
IFFO2	CKMT2	C19orf33	CENPT		PTPRZ1
DNAH7	CCDC24	DSG3	DYRK2		TNPO1
KDM6B	ANKRD20A9P	RNF19A	SLC12A4		FAM135A
EGR3	PLAUR	ZNF592	FKRP		WWC2
CCDC165	ZNF236	AFAP1	TSC2		SIK2
ANO9	KIFC2	ANKRD36B	ZNF646		GNAS
ANKRD18A	KIAA1804	LOC647859	ZNF503		PLSCR1
GRHL2	BAGE4	ZBTB10	HSPD1		TRPC4AP
IER5	HMGA1	LLGL2	USP11		RPL15
BAGE5	PTPRD	KIAA1530	PTBP2		PREX2
TRPV6	SIPA1L3	RBM15	PAN2		KCNMA1
H1FX-AS1	PTPN13	NPIPL3	PRKD2		DPP3
SOX4	OTUD3	MAP2	FAM60A		ARL13B
LAMB3	AGRN	UBE2S	NXF1		MBTPS1
PRDM15	SPATA5	PFAS	KIF1B		AKAP13
KIAA0284	PER2	LOC595101	CRTC2		VEPH1
C6orf132	LAMC1	ZKSCAN2	ANXA11		TMEM229B
LOC646851	TBC1D3H	TBC1D8	ASH1L		BRCC3
HUNK	CLIC6	CDR2L	HNRNPH1		EIF2S1
WDR27	MCL1	TNFRSF10A	ARGLU1		FAR2
HSPB8	TMEM212	BRD4	NUFIP2		USP25
TMPRSS4	MFI2	PTPN14	DHX9		MALL
PLXNB3	SMOX	C12orf75	SCAF8		RNF141
LDLR	SEMA3E	OTUD1	BDP1		UTRN
EPS8L1	LOC113230	FNBP1L	EXOC8		INSR
GOLGA6L1	NRARP	RNF19B	SON		TTC3
LOC100131366	ESRRG	LOC729737	ODF2L		UBD
FGF9	SPC25	PLEKHM1P	GPATCH8		TIMP3
CLDN9	EZR	PKD1P1	ABLIM1		PIGU
N4BP3	IER2	SMURF1	RBM19		GRB10
MBOAT4	OPHN1	TMEM184A	SAMD12		MARVELD3
GGT6	CUEDC1	SUGP2	GPD2		SAMD10
BIK	ProSAPiP1	KIAA0913	PRKCD		NDST1
KLHL21	FLJ13197	NAA16	EFNB1		NRDC
TMEM158	NAALADL2	CSNK1G1	KLHL25		TMPO
FAM83G	FILIP1L	FAM189B	LONRF1		USP12
KLF6	ANXA3	PABPC1L	LEMD3		F2RL1
KIF12	LOC643406	MAP3K1	RC3H1		MED13
C16orf93	FGFR3	NEO1	RBM39		BRD7
C20orf54	MYADM	RBM38	PI4KAP2		EIF3E
MAFF	NPTX2	CAD	S100A11		ESYT1
TMTC2	ARHGAP8	KIF13B	UBAP2		OSBPL9
FA2H	LOC100216545	LONRF2	MPRIP		PPP2R1B
ARHGEF38	SULT1C4	ZFAND2A	NPEPPS		PKP2
FOXQ1	KIAA0664L3	KANSL1	RBM14		STAT3
USP43	TP53BP1	BRD2	VEZF1		CPNE8
NME5	SQLE	RALGAPA1	H3F3C		TERF2IP
ARNT2	DEDD2	ZFC3H1	PTPRM		ITGAV
BBC3	GOLGA8B	INPP5E	SPTAN1		IL1RN
EPHA2	EFCAB4A	AGAP6	DMWD		HDAC10
HSPB1	MLLT4	TTLL4	ZBTB5		UMPS
B3GNT5	LDOC1	IRF1	USPL1		CCL2
ITGA3	ITPR3	HSP90AB3P	CDKN2AIP		RRAGD
BZRAP1	IER3	C9orf117	PNN		IFNAR1
SLC35F2	PRSS8	KAT2A	BRD1		PSEN1
PCDH11Y	PPP1R13B	BOD1L	KAT6B		CTPS2
DUSP8	LOC401052	SLC35E4	ZNF600		ARHGEF10
C11orf9	INHBA	EIF2C2	FOXP1		GATAD2B
BCL2L15	SPAG1	SESN2	HNRNPA0		DOCK1
DUSP2	VGLL3	MAP1S	VPS37C		NDUFA10
SLC7A5P1	AP1G2	UPF1	H1FX		NT5C3A
LOC440894	PLEKHN1	MYH9	CCNL2		TP53INP2
WEE1	LOC150776	SH2B2	RSRC2		GFPT1
CDKN2B	C7orf46	CMTM7	NBPF24		LTBP2
TPPP	SLC7A5	TSPAN15	CABIN1		IRAK1BP1
TP53BP2	PLEKHA7	DSC2	TOMM34		SMPD3
CSRNP1	MYLIP	KDM6A	CHD1		ATAD1
FAM46B	PGM5P2	ZNF692	BTG1		SPTLC2
LOC284454	CX3CL1	LOC641298	HMGXB3		C19orf53
BMP2	CLK1	PDP1	CARD10		STRBP
SGPP2	CLDN3	SEL1L3	SNHG12		VCAM1
DUSP4	SH2D3A	TAF1D	MLXIP		AMBRA1
POM121L8P	COL27A1	PRR3	LOC389906		PAIP1
COL9A2	RAI1	PYGB	FOXO3		RAP2A
DSC3	EPHA10	LOC100132247	C10orf118		SLA2
MAFK	BAMBI	ADRA2C	PIP5K1A		PRKCE
MXD1	CLDN10	BRAF	INO80		PPP1R14B
HERC2P4	TRIM16	RASSF8	LOC440434		UBR5
B4GALNT3	EPHB6	RGNEF	MLL3		COMMD5
ATP8A2	RUSC2	ADAM9	C3orf58		ILDR1
ITPKC	IFT140	GOLGA8A	EFNB2		TMEM45B
TBC1D3C	FOSL2	ZNF407	USP42		SEC62
TRIB1	JAG1	JUP	SP4		MANSC1
NFKBIA	ZNF711	MEX3C	ASXL1		BMPR2
LOC386597	LAD1	CDC42BPB	ZSCAN18		SIAH1
EDN2	KIAA1244	PCF11	MED15		C1QL3
LAMA1	CHD3	C12orf51	CDK16		CGN
ESRP1	C17orf28	SLC3A2	BEND7		TOM1L1
DFNB31	RPL23AP64	REXO1	BAHD1		RHOBTB3
LOC100127888	MALAT1	IRF6	ARID2		PLD1
C19orf35	TNFRSF21	CDKN1C	HCG18		SLMAP
KAAG1	GYLTL1B	KCNC4	TCERG1		MRPL14
CYR61	ASPHD1	MCF2L	FUS		ZBTB33
TFPI2	MPP3	KBTBD8	PLEC		LIN7C
NCEH1	SHANK2	NPIP	MLL5		CDCA3
TPPP3	NPNT	SRCIN1	FAM193A		C6orf62
TMPRSS3	ZNF483	CAPN12	MED12		ATP5IF1
TMCC2	ITGA2	RLF	URGCP		SF3B1
KRT80	TBC1D22B	SPPL2B	CEP250		SCX
TPBG	KIAA0754	PIM3	LUZP1		IFT57
MYEF2	ZBTB20	SDHAP1	NUP188		KCTD3
RAB11FIP1	KCNQ1OT1	FZD7	CNOT4		ARF3
PPL	HES4	STIP1	WHSC2		CHERP
PIWIL4	MDC1	IL28RA	NIPBL		PSMD7
B7H6	KDM5B	FCHO1	PPM1D		PAK2
STOX2	SLC16A7	NFYA	WHAMM		CSRP1
PHLDA2	PARD6B	EP300	THRAP3		IGSF11
LAMA5	FBXL18	HEXIM1	TBC1D19		NEK5
FAM46A	MAP3K14	YLPM1	WIPF2		CYHR1
CCDC64B	TRIO	RHBDF2	PLP2		RBPMS
TSC22D2	LOC100131257	TRIP10	SAFB2		TMEM63B
HSP90AA1	HIVEP2	NUMA1	ZNF317		LRP5L
LOC388152	CAMSAP1	BMS1P1	DOPEY1		SPRR2G
TLL1	SLX4	GON4L	MYSM1		FOXJ2
C15orf52	CHD2	LOC100499177	PMS2P3		LZTR1
BIRC3	ST14	FGD6	SMG1		PPP2CB
CCDC141	SPEN	WWTR1	TC2N		TWF1
GRB7	REL	CBL	LMTK2		TGFBR2
LOC100652770	PKD1	PPM1L	NFRKB		PLXNA1
KRT86	CNNM4	MPZL3	NCKAP5L		GSK3B
KIAA1522	LARP6	TOB2	EPC1		KLHDC7A
ICA1L	SRF	MCAM	FADS3		ZMYM2
LOC100499467	PLEKHA8P1	TJP1	SMG7		PHACTR4
ZSCAN12P1	IGF2BP2	MZF1	SFSWAP		LCN2
TRIM45	ZBTB34	EDC4	SCAF4		PROX1
ZNF296	INADL	CYTH2	AFTPH		PSMC6
PLK2	MAPK8IP3	HNRNPU	SRRM1		MLPH
PLEKHB1	ARHGAP39	AP1S3	HSPA4		TUBA1C
EPS8L3	FAM53C	TNKS1BP1	RNF38		CKMT1B
LOC643650	HIP1R	LRRC1	TRIM33		RBM5
NCOA7	RIPK2	TSC1	CTBP2		CLIP1
GALNT3	HSP90AB2P	PLEKHG3	KIAA1967		TCF7L2
PROM1	LOC91948	RAB11FIP4	PTBP1		TBL1XR1
GDF9	MAMLD1	PER1	SAMD4B		MYOF
AMOTL2	LMNA	KIF3A	H3F3B		PIK3AP1
BCL2	SERTAD2	PCNT	WAPAL		SHISA4
KLF4	ZNF217	RGPD5	GPBP1		ELK3
SLC12A2	LIPH	MCOLN3	ATAD2B		ING4
HIST2H2BE	FLNB	SF3A2	MTMR3		MAP2K7
IL17RD	NPHP4	C2CD3	SWAP70		COPZ1
MFSD6L	SLC4A4	CSF1	PM20D2		FAM149A
DIDO1	ARID4B	CSNK1E	FOXP4		
CKAP5	TRMT1	USP24	B3GNT2		
ZNF384	MFSD10	SIN3A	POM121C		
AMMECR1L	SUPT6H	DCAF5	STRN		
SGSM2	AAK1	ANKHD1			
